# A comprehensive study of intratumor bacteria in clear-cell renal cell carcinoma identifies *Cutibacterium acnes* as a protector via suppression of macrophage M2 polarization

**DOI:** 10.1128/msystems.00500-26

**Published:** 2026-07-15

**Authors:** Yuqing Li, Dengwei Zhang, Linyi Tan, Junyao Xu, Yang Sun, Rui Zhang, Yao Cheng, Yong-xin Li, Ting Guo, Haowen Jiang, Wei Zhai, Chenchen Feng

**Affiliations:** 1 Department of Urology, Huashan Hospital, Fudan University 159397, Shanghai, China; 2 Department of Chemistry and The Swire Institute of Marine Science, The University of Hong Kong, Hong Kong SAR, China; 3 Southern Marine Science and Engineering Guangdong Laboratory (Guangzhou) 606379 , Guangzhou, China; 4 State Key Laboratory of Oncogenes and Related Genes, Department of Urology, Renji Hospital, School of Medicine, Shanghai Jiao Tong University 12474, Shanghai, China; 5 Cancer Institute, Xuzhou Medical University 38044, Xuzhou, Jiangsu, China; 6 Shanghai KR Pharmtech, Inc., Ltd, Shanghai, China; 7 Department of Gynecology, Obstetrics & Gynecology Hospital of Fudan University, Shanghai Key Lab of Reproduction and Development, Shanghai Key Lab of Female Reproductive Endocrine Related Diseases 92276, Shanghai, China; Qingdao Institute of Bioenergy and Bioprocess Technology, Qingdao, Shandong, China

**Keywords:** intratumor bacteria, clear-cell renal cell carcinoma, prognosis, *Cutibacterium acnes*, macrophage polarization

## Abstract

Intratumor bacteria (ITB) have emerged as key modulators in various malignancies; however, their role in clear-cell renal cell carcinoma (ccRCC) remains unclear and elusive. In this study, we analyzed seven independent cohorts (comprising one 16S rRNA sequencing and six bulk RNA sequencing data sets) encompassing 517 ccRCC and 65 paired adjacent normal tissues. Following a rigorous decontamination pipeline, we found that neither microbial diversity nor compositional profiles differed significantly between cancerous and adjacent normal tissues. Thirteen specific genera—including *Streptococcus*, *Rothia*, *Cutibacterium*, and *Corynebacterium*—exhibited significant prognostic associations. The microbial risk scores constructed from these genera demonstrated robust performance in prognostic risk stratification and were validated as an independent prognostic factor for both overall survival and progression-free survival, respectively. Host-microbe interaction analysis revealed correlations between these prognostic genera and oncogenic pathways, particularly those involved in immune regulation and metabolism. Notably, culturomics successfully isolated *Cutibacterium acnes* (*C. acnes*) from ccRCC tissues, and higher *C. acnes'* abundance was correlated with a favorable prognosis. Functional assays revealed that *C. acnes* inhibits tumor growth and suppresses macrophage M2 polarization. Mechanistically, *C. acnes* and its primary metabolite, propionic acid, attenuate macrophage M2 polarization by activating TNF signaling. Our findings underscore the protective role of *C. acnes* in the ccRCC microenvironment and highlight its potential as a target for precision immunotherapy.

**IMPORTANCE:**

To address previous methodological limitations, we applied rigorous decontamination to multi-omic datasets to isolate and experimentally validate intratumor bacteria colonization in ccRCC. Comparable profiles between tumorous and normal tissues suggest intratumor bacteria generally act as “passengers.” We successfully constructed robust microbial risk scores predictive of patient survival. Mechanistically, *in vitro* and *in vivo* models demonstrated that *Cutibacterium acnes* and its metabolite, propionic acid, inhibit tumor growth and suppress M2 macrophage polarization via TNF signaling. These findings highlight the protective role of *Cutibacterium acnes* in the tumor microenvironment, revealing it as a promising target for precision immunotherapy.

## INTRODUCTION

Along with the development of the sequencing technologies, the intratumor microorganisms, including bacteria (intratumor bacteria, ITB), fungi, and viruses, have been observed as a crucial component of the tumor microenvironment and have been proved to exist widely in a variety of tumors such as gastric cancer (1), colorectal cancer (2), hepatocellular cancer (3), etc., establishing microbiome as a novel omics or “second genome” of cancer (4). Although the studies about the connection between microbiome and cancers, for example, the human papillomavirus and the cervical cancer (5), had been revealed early, it was not until Nejman et al. (6) published that microorganisms were resident in multiple solid tumors (breast, lung, ovarian, pancreatic, melanoma, bone, and brain) that intratumor bacteria began to attract widespread attention. The intratumor bacteria have been documented to be closely associated with cancer initiation, progression, metastasis, and treatment outcomes by multiple ways, including causing DNA damage, inducing the activation of proinflammatory responses and other oncogenic pathways, inducing immunosuppression, and metabolic reprogramming (7). Therefore, further investigation into the role of intratumor bacteria in tumors is of great significance.

Clear-cell renal cell carcinoma (ccRCC) is the most common type of malignancy in the kidney (8) that is conventionally accepted as a sterile organ, and ccRCC is expected to harbor a low biomass of microbiome. Early researchers revealed the presence of tumor tissue-resident microbiota in renal cell carcinoma and recognized the difference in microbial composition and specific taxa between cancer and normal tissues (9, 10). The tumor-resident bacteria may also serve as a noninvasive predictor of PD-L1 status in venous thrombi (11). Viruses, such as human papillomavirus and Epstein-Barr virus, have also been detected in RCC tumor tissues and were found to be correlated with higher-grade tumors (12, 13). Besides, the intratumor mycobiome was also quantitatively profiled and showed potential to predict prognosis and immunotherapy outcomes (14). Although pieces of research on intratumor microbes in renal cell carcinoma have made some progress, the reliability of the results remains questionable due to insufficient sample size and the lack of systematic methods (15), especially for the systemic study focusing on intratumor bacteria within ccRCC tissues.

In this study, we integrated multiple cohorts and multiomics data sets, as well as culturomics and experimental validation, to investigate the presence of the intratumor bacteria and the impact of that on the tumor microenvironment, prognosis, and carcinogenic pathways in patients with ccRCC.

## MATERIALS AND METHODS

### Study population

We incorporated seven independent data sets into our study, as summarized in Table S1. For 16S rRNA sequencing, we retrospectively collected 217 tumor and 27 normal samples from 217 patients who had been histologically diagnosed with ccRCC and underwent partial or radical nephrectomy in Huashan Hospital (Shanghai, China) between May 2013 and Oct 2022 under reasonable inclusion criteria. No subjects received preoperative treatments, including immunotherapies or molecular targeted therapies. The samples were formalin-fixed and paraffin-embedded (FFPE). We also included 10 negative controls using sliced paraffin from the margin of the block, sampling paraffin only without tissue.

A total of six cohorts with the bulk RNA sequencing data available were included in our study. EGAD00001000597 (16) is an integrated molecular study of ccRCC and consists of 100 tumor samples. EGAD00001006029 (CheckMate 025; NCT01668784) (17) is a prospective clinical trial of the anti-PD-1 antibody nivolumab in advanced ccRCC, and 53 tumor tissues were obtained prior to initial therapy for patients enrolled in this study, including 14 patients with an objective response record of immunotherapy. Data from the above two data sets were requested from the principal strictly via the European Genome-phenome Archive (EGA; https://ega-archive.org/) according to the clinical data transfer agreement. Another three data sets from the GEO database, under accession numbers GSE102101 (18), GSE126964 (19), and GSE151419 (20), containing both the tumor and adjacent normal samples of renal cell carcinoma, which were organized by Singapore, China, and Poland, respectively, were also included, and the raw sequencing data were downloaded from the Gene Expression Omnibus (GEO) data repository. The Renji cohort contained 27 ccRCC patients who underwent nephrectomy and did not receive any therapy before surgery, and the RNA-seq data were supplied by Renji Hospital (Shanghai, China).

For improved readability and to avoid potential confusion between similarly named cohorts, we implemented a standardized numbering convention where the EGAD00001000597 data set is identified as Cohort 1, EGAD00001006029 as Cohort 2, GSE102101 as Cohort 3, GSE126964 as Cohort 4, GSE151419 as Cohort 5, and Renji as Cohort 6 throughout all analyses.

Additionally, one single-cell RNA sequencing (scRNA-seq) data set was also retrieved from the GEO database under accession number GSE171306. This data set includes scRNA-seq data from two bilateral ccRCC tumor tissues.

### 16S rRNA gene amplicon sequencing

In preparation for the 16S rRNA gene sequencing, samples were sectioned from the paraffin-embedded tissue blocks, which underwent quality testing, purification, and nested amplification. To meet the requirement of sufficient DNA for sequencing, the amplified products were detected by DNA electrophoresis, and the eligible samples were kept for further study. 16S rRNA gene sequencing was conducted at the Nonogene Co., Ltd. In brief, genomic DNA was extracted from the tissue samples using the CTAB/SDS method. The 16S rDNA V4 region was amplified through PCR employing a primer pair (515F: 5′-GTGCCAGCMGCCGCGGTAA-3′ and 806R: 5′-GGACTACHVGGGTWTCTAAT-3′) with a barcode. Sequencing libraries were prepared using the TruSeq DNA PCR-Free Sample Preparation Kit (Illumina, USA) following the manufacturer’s instructions. The libraries were subsequently sequenced on the Illumina NovaSeq platform, yielding 250 bp paired-end reads.

### Isolation of intratumor bacteria

Multiple ccRCC and adjacent normal tissue blocks were subjected to a variety of aerobic and anaerobic agar plates for potential ITB isolation. Tissue blocks were carefully excised. After homogenizing thoroughly with a homogenizer pestle in 1 mL of PBS solution at 4°C, the mixture was shaken for 20 min at a speed of 380 rpm at 15°C. The supernatant was diluted 10 times with PBS, and a sample (100 μL) was inoculated onto an agar plate. Multiple environmental controls were taken at the time of procedure. At 2 weeks, only one anaerobic plate showed five clones, and none was noted in any other plate or any of the controls. 16S rRNA sequencing identified that all five clones were *C. acnes*.

Extended methods are detailed in the Supplemental text.

## RESULTS

### The landscape of ITB in ccRCC

**Fig 1:**
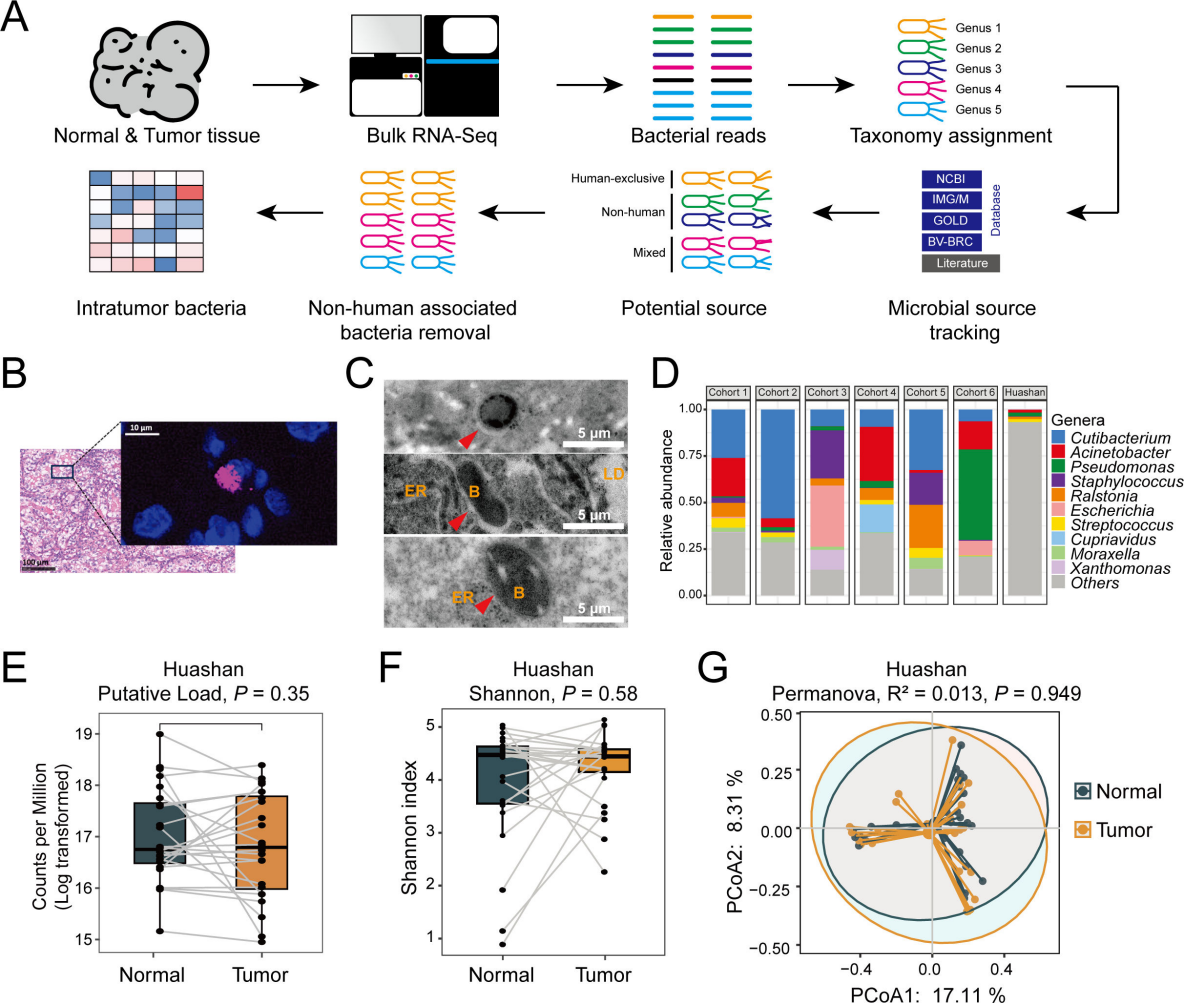
The landscape of intratumor bacteria in ccRCC. (**A**) Schematic diagram illustrating the process of analyzing bulk RNA-seq data to identify intratumor bacteria. The analysis involves using bulk RNA-seq data from normal and tumor tissues. To track potential microbial sources, source annotations from bacterial genomes in databases such as NCBI, IMG/M, GOLD, and BV-BRC, as well as literature search, are retrieved. Bacteria associated with the human host are retained for constructing the intratumor bacterial matrix. (**B**) Representative images of fluorescence *in situ* hybridization (FISH) staining of 16S rRNA in tumor tissues of ccRCC. (**C**) Representative TEM images showing the presence of bacteria-like structures with cell walls and characteristic nucleoid regions within the ccRCC tumor microenvironment. The red arrow indicates the intratumor bacterium. B: bacteria; ER: endoplasmic reticulum; LD: lipid droplet. Scale bar, 5 μm. (**D**) Stacked bar plot showing the proportion of genera present in at least five cohorts among the seven cohorts. (**E and F**) Box plot showing the difference of putative load (bacterial counts per million reads) and Shannon index between 24 paired tumor and normal samples in the Huashan cohort. The *P*-value was given by the paired Wilcoxon rank-sum test. (**G**) Principal coordinate analysis (PCoA) of 24 paired tumor and normal samples in the Huashan cohort, based on the Bray-Curtis dissimilarity. The *P*-values were tested by permutational multivariate analysis of variance (PERMANOVA).

Following the analytical workflow (Fig. S1), we established a comprehensive atlas of the ITB in ccRCC. Given the low-biomass nature of these clinical specimens, we implemented a stringent and tailored decontamination algorithm (Fig. 1A) to discern authentic microbial signals from pervasive sequencing noise. The overall ITB abundance was low in ccRCC, corresponding to the notion of a low biomass nature, with 40% primarily identified as contaminant genera (Fig. S2). Raw ITB reads took up ~(1/2.00E−06) of total sequencing reads (Fig. S3A) and showed a positive correlation with total reads (Fig. S3B); 327 out of 545 genera survived after decontamination (Fig. S3C). Our passes not only managed to filter out non-human reads (Fig. S3D) but also showed an increase in the proportion of common contaminants after decontamination, indicating that some bacteria, previously accepted as contaminants, could be indwelling in ccRCC (Fig. S3E). The relative abundance of non-human-associated bacteria dropped consistently in all cohorts following our decontamination (Fig. S3F). Common genera across RNA-seq cohorts after decontamination remained comparable either grouped by data set or by sample (Fig. S4A through D). Parallel 16S rRNA sequencing further authenticated the intra-tissue localization of these signals, revealing comparable read-reduction patterns in both malignant and adjacent normal tissues (Fig. S4E and F). While the microbial composition exhibited inherent inter-cohort heterogeneity (Fig. S5A through D), *Proteobacteria* and *Firmicutes* emerged as the predominant phyla across all bulk and single-cell RNA sequencing (scRNA-seq) data sets (Fig. S5A and S6; Table S2). Intriguingly, microbial reads were virtually absent within high-quality host cells in the scRNA-seq data (Fig. S6). The 16S rRNA-targeted FISH probe to tissue blocks showed the ITB existence in ccRCC (Fig. 1B). High-resolution electron microscopy (EM) analysis revealed that bacteria-like structures were present in 6.67% (8/120) of the fields of view across ccRCC tissue sections. Furthermore, these structures were distributed within both the intracellular and extracellular compartments; specifically, 70.83% (*N* = 17) were localized within the cytosol, whereas 29.17% (*N* = 7) resided in the extracellular space (Fig. 1C). By cross-referencing the top 20 most abundant genera, we identified 11 genera present in over five cohorts, with *Cutibacterium* uniquely persisting across all cohorts (Fig. 1D; Table S3). Herein, we concluded that ccRCC harbored a low biomass of ITB and identified the presence and composition of ITB in ccRCC.

### ITB does not differ between cancer and adjacent normal tissue in ccRCC

Using decontaminated reads (Table S4), we next characterized ITB across three dimensions: putative ITB load, bacteriome diversity and composition, and individual ITB features. The putative ITB load did not differ between paired tumor and normal tissues across all cohorts (Fig. 1E; Fig. S7A). Likewise, neither α-diversity nor β-diversity showed significant differences between normal and cancer tissues (Fig. 1F and G; Fig. S7B through D). We further investigated whether individual ITB genera were differentially distributed in a reproducible manner. Consistent with the lack of significant differences identified by the Wilcoxon test (Fig. S8A), although random forest analysis initially nominated 10 candidate differential ITB genera (Fig. S8B and C), this machine-learning approach could hardly validate these features with satisfactory predictive performance on distinguishing between tumor and normal samples across cohorts (Fig. S8D). Collectively, these results indicate that, in contrast to observations reported in many other tumor types, a differential bacteriome between ccRCC tumors and adjacent normal tissues may be absent in ccRCC. Our findings suggest that most ITB genera detected in ccRCC likely represent inherent intra-tissue bacteria residing in the kidney, implying a passenger rather than a driver role of ITB during the tumorigenesis stage of the tumor (21, 22).

### ITB risk score predicts prognosis in ccRCC

**Fig 2:**
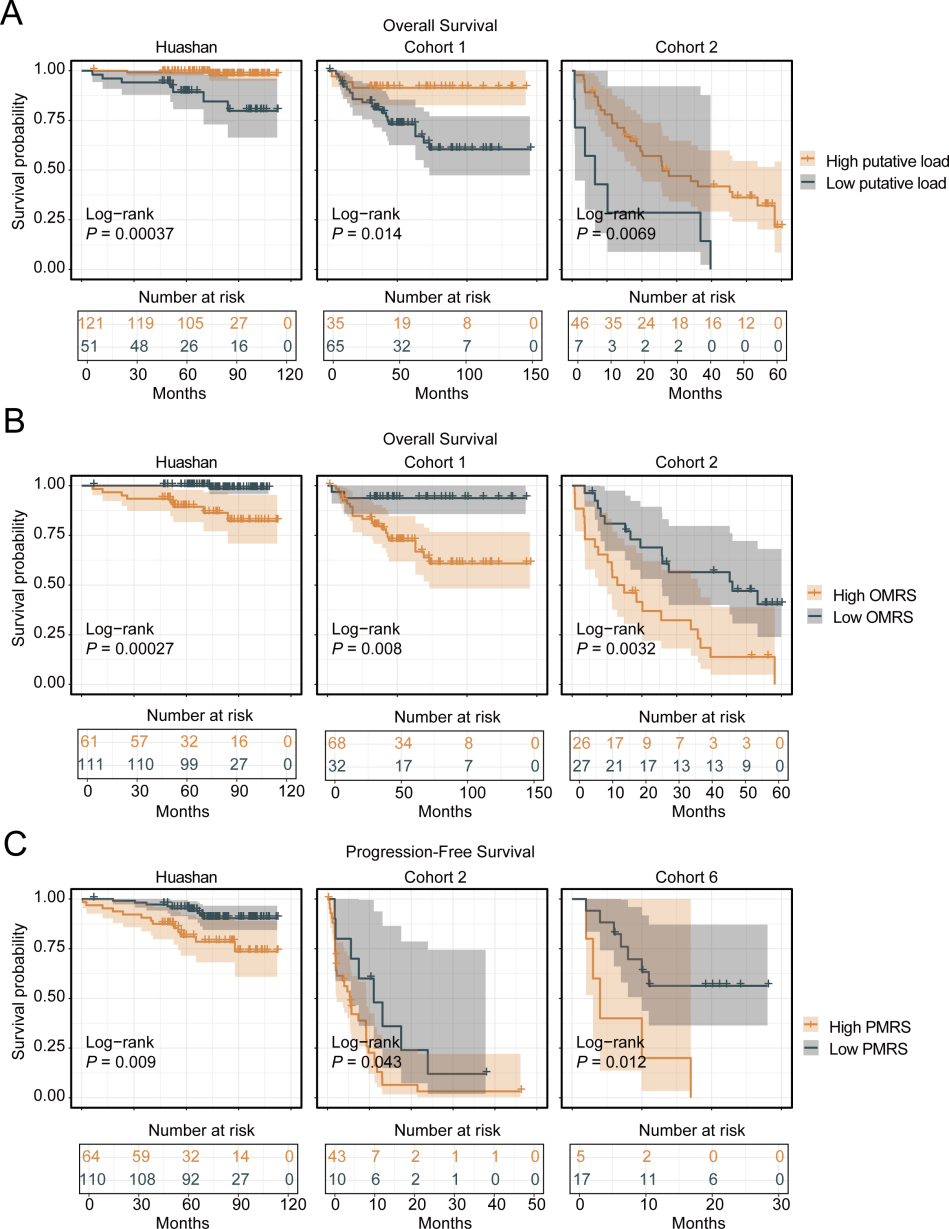
Putative intratumor bacteria load and microbial risk score predict prognosis in ccRCC. (**A**) Kaplan-Meier curves showing the OS probability stratified by the putative ITB load across cohorts. (**B and C**) Kaplan-Meier curves showing the OS probability and PFS probability of patients stratified by the OMRS and PMRS across cohorts, respectively. The *P*-values were calculated using an unadjusted log-rank test. ITB, intratumor bacteria; OS, overall survival; PFS, progression-free survival.

Correlation analysis revealed that the ITB signature was independent of conventional clinicopathological variables. Specifically, no significant links were identified with parameters such as age, tumor stage, or grade in any of the cohorts studied (Fig. S9). Likewise, no apparent linear correlation was observed between intratumor microbial composition and patient prognosis across cohorts (Fig. S10A and B), but distinct microbial signatures remained evident within specific cohort-subgroups in terms of both overall survival (OS) and progression-free survival (PFS) (Fig. S10C and D), underscoring the potential of the intratumor microbiome as a novel prognostic biomarker. We found that a higher computationally estimated microbial burden was predictive of favorable OS in all cohorts (Fig. 2A) and enhanced PFS in two of the three cohorts (Fig. S11). Given the computational nature of these estimates, rigorous PCR-based validation remains warranted. Subsequent screening using univariate Cox proportional hazards regression across the three cohorts yielded 13 distinct microbial genera significantly linked to patient prognosis (Fig. S12). Derived from these taxa, we formulated dual microbial risk scores (MRSs): the OMRS for OS and the PMRS for PFS. These scoring systems not only achieved robust patient stratification but were also validated as independent prognostic factors for their respective survival metrics (Fig. 2B and C; Tables S5 and S6). Among these features, *Streptococcus*, *Rothia*, *Cutibacterium*, *Acinetobacter*, *Actinomyces*, and *Corynebacterium* demonstrated elevated prevalence and relative abundance (Fig. S13A and B). These taxa were further identified as hub nodes within the correlation network, distinguished by high degree and betweenness centralities (Fig. S14A and B), which underscores their dominant roles in preserving the microbial community structure.

### ITB might affect host metabolic and immune pathways in ccRCC

**Fig 3:**
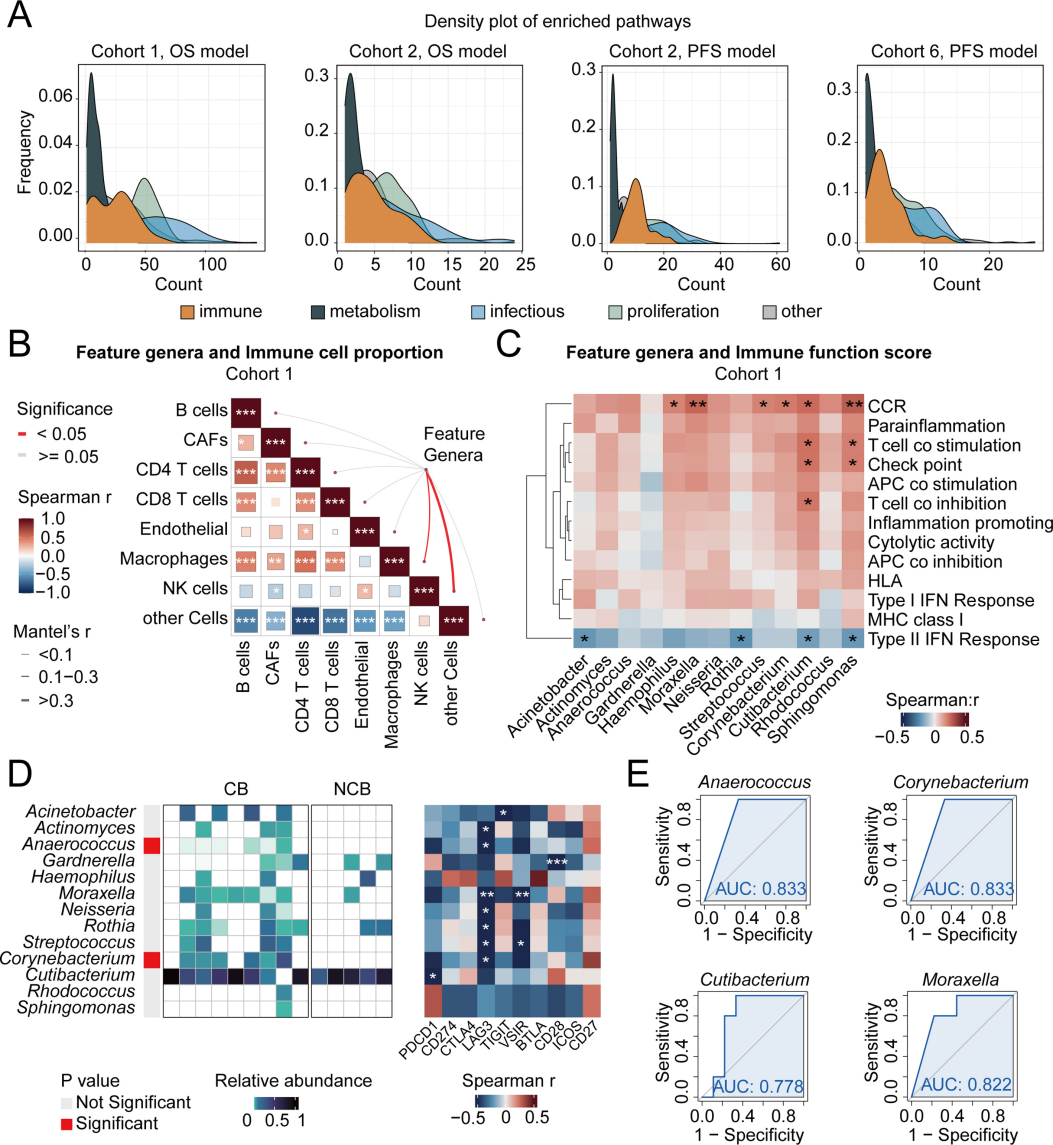
Intratumor bacteria and host gene interactions in ccRCC. (**A**) The density curves represent the distribution of the pathways that were significantly enriched by KEGG analysis. The horizontal axis represents the count of the genes hit in the pathway, while the vertical axis represents the frequency. The differentially expressed genes used for enrichment analysis were identified between subgroups stratified by the OMRS in cohort 1 and 2, and the PMRS in cohorts 2 and 6, respectively. (**B**) Heatmap showing the association between the distance matrices of feature genera and of the immune cell proportion in tumor tissues in the Huashan cohort, and the Mantel test gave the *P*-value. The connection line colored red implied significant link, and the width of the line implied the absolute value of Mantel’s *r*. The association between immune cell proportion was calculated by the Spearman test. (**C**) Heatmap showing the association between the feature genera abundance and immune function score in tumor tissues in the Huashan cohort. The Spearman test gave the *P*-value. (**D**) The heatmap at left shows the relative abundance of the differentially enriched genera between patients with clinical benefit (CB) and non-clinical benefit (NCB) of ICB. The χ² test gave the *P*-value. The heatmap at the right indicated the correlation between feature genera abundance and mRNA expression of immune checkpoint genes, including PDCD1, CD274, CTLA4, LAG3, and so on. Spearman gave the *P*-value. ICB, immune checkpoint blockage. (**E**) Receiver operating curves of abundance of genera, including *Anaerococcus, Corynebacterium, Cutibacterium,* and *Moraxella* as predictive of response to ICB in cohort 3, and the area under curves (AUC) suggest the predictive capability of each genus. The significance was shown with asterisks. *P < 0.05; **P < 0.01; ***P < 0.001.

Accumulating evidence indicates that intratumor microbiota regulate cancer progression via crosstalk with tumor immunity, modulation of cancer metabolism, and affecting other cellular signaling pathways (23), which necessitate the integration of transcriptomic profiling to elucidate the mechanisms by which microbial signatures modulate host biological regulation in ccRCC. Functional enrichment analysis revealed that differentially expressed genes (DEGs) between high- and low-risk groups, stratified by either OMRS or PMRS, were primarily concentrated in immune- and metabolism-related pathways (Fig. 3A; Tables S7 to S10). These findings strongly suggest a pivotal link between the feature genera and host immune-metabolic homeostasis. Although prognosis-associated ITB showed a broad association with immune infiltrates and immune-related signaling, they tended to play a role as a whole as host interactions of each feature were weak, reflecting the low biomass nature (Fig. 3B and C; Fig. S15A and B). In general, CD4^+^ T cells and macrophages showed relatively extensive association with ITB features (Fig. S16). We next examined the microbial profile associated with immune checkpoint blockade (ICB) and identified an enrichment of *Anaerococcus* and *Corynebacterium* in ccRCC patients who achieved a complete benefit (CB) from Nivolumab (Fig. 3D). Interestingly, both genera exhibited significant interactions with LAG3 expression (Fig. 3D). Beyond these two taxa, *Cutibacterium* and *Moraxella* also demonstrated a strong capacity for predicting clinical responses to immunotherapy (Fig. 3E). Taken together, our findings suggest that ITB are closely associated with host immunity and metabolic pathways—two axes we will investigate further in subsequent mechanistic studies.

### *C. acnes* is present in ccRCC and acts in a protective role

**Fig 4:**
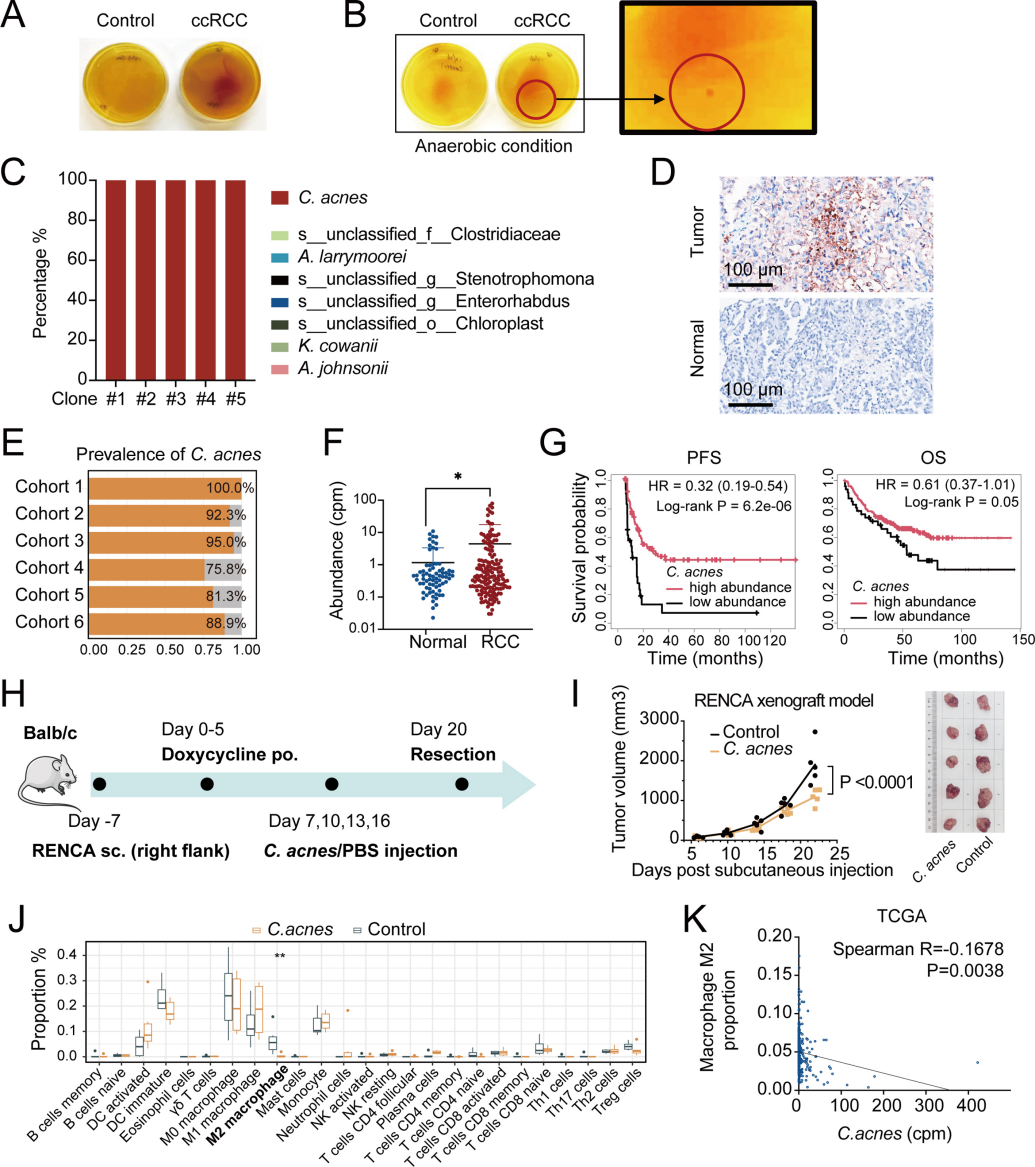
*C. acnes* is present in ccRCC and acts in a protective role. (**A and B**) Culturomics of ccRCC tissue blocks under aerobic and anaerobic settings. (**C**) Five clones collected from anaerobic cultures all revealed *C. acnes* identified by 16S rRNA sequencing. (**D**) Representative images of immunohistochemistry staining using *C. acnes* antibody on ccRCC and normal samples. Scale bar, 100μm. (**E**) Prevalence of *C. acnes* across RNA-seq cohorts. Retrieved from all RNA-seq cohorts and aggregated at the species level, shown were (**F**) abundance (CPM format) of *C. acnes* between cancerous and adjacent normal tissue, and (**G**) stratification ability of *C. acnes* abundance for OS and PFS, respectively. The *P*-values were calculated using unadjusted log-rank test. OS, overall survival; PFS, progression-free survival. (**H**) RENCA cell murine xenograft model was constructed, followed by doxycycline administered intragastrically and intratumor injection of *C. acnes*. The tumor size was recorded. When the tumor size exceeded 2,000 mm^3^, the mouse was euthanized. (**I**) Shown was the change of tumor volumes between *C. acnes*-treated and control groups, and tumor images at the observation endpoint. The tumor sizes at the endpoint between groups were compared using the Wilcoxon rank-sum test. (**J**) The proportion of 22 infiltrating tumor cells evaluated by computational approach from the transcriptome of the above murine models between the control and *C. acnes*-treated group. The *P*-value was given by Wilcoxon rank-sum test. (**K**) The correlation between *C. acnes* abundance and M2 macrophage proportion in the TCGA-KIRC data set. The Spearman test provided the *P*-value. The significance was shown with asterisks. *P < 0.05; **P < 0.01.

Culturomics with various controls were performed using 10 fresh samples from ccRCC patients. With careful operations, cultures in most samples yielded no growth except for contaminants (Fig. 4A). We obtained bacterial clones in one sample, whereas no bacteria grew in any control condition in an anaerobic setting (Fig. 4B). 16S rRNA sequencing of all five clones showed predominant abundance of *C. acnes* (also named as *Propionibacterium acnes*) (Fig. 4C), and the whole-genome sequencing identified the strains as subtype II, which was also isolated and identified highly metabolically active colonizing strains in deep non-cutaneous tissues, such as solid tumors, including prostate and breast cancers (24). Although *C. acnes* is considered contamination in some studies (25), we consider our culture solid, as none of the controls had any bacteria grow, and *C. acnes* is among the top abundant microorganisms in ccRCC that is prone to be cultured in comparison to other species with lower biomass. Also, *C. acnes* could also be identified positive in 12/20 ccRCC paraffin samples using immunohistochemical staining (Fig. 4D). In our study, *C. acnes* was revealed in 75%–100% tumor samples across RNA-seq cohorts (Fig. 4E), and tumors harbored higher *C. acnes* abundance than adjacent normal tissues did when all ccRCC samples were combined and normalized (Fig. 4F). However, a higher abundance of *C. acnes* in ccRCC was associated with significantly better PFS and a marginal improvement in OS (Fig. 4G), indicating a potential driver and protective effect of *C. acnes*.

To explore the effect of *C. acnes* on ccRCC progression, we conducted a phenotypic experiment using the RENCA xenograft mouse model, which showed inhibition of renal tumor growth following intratumor injection of *C. acnes* (Fig. 4H and I). We co-cultured ccRCC cells with *C. acnes* and found no change in proliferation after 60 h (Fig. S17). We thus postulated that *C. acnes* could impact tumor-infiltrating lymphocytes (TILs). Tumors from the animal model above were subjected to transcriptome analysis, and TILs were assessed by a computational immune profiling approach. Of note, M2 macrophage was significantly decreased in the *C. acnes-*treated group (Fig. 4J). Correlation analysis in the public data set also showed a significant negative correlation between *C. acnes* abundance and M2-polarized macrophage (Fig. 4K).

### *C. acnes* and propionic acid stimulate TNF to suppress macrophage M2 polarization

**Fig 5:**
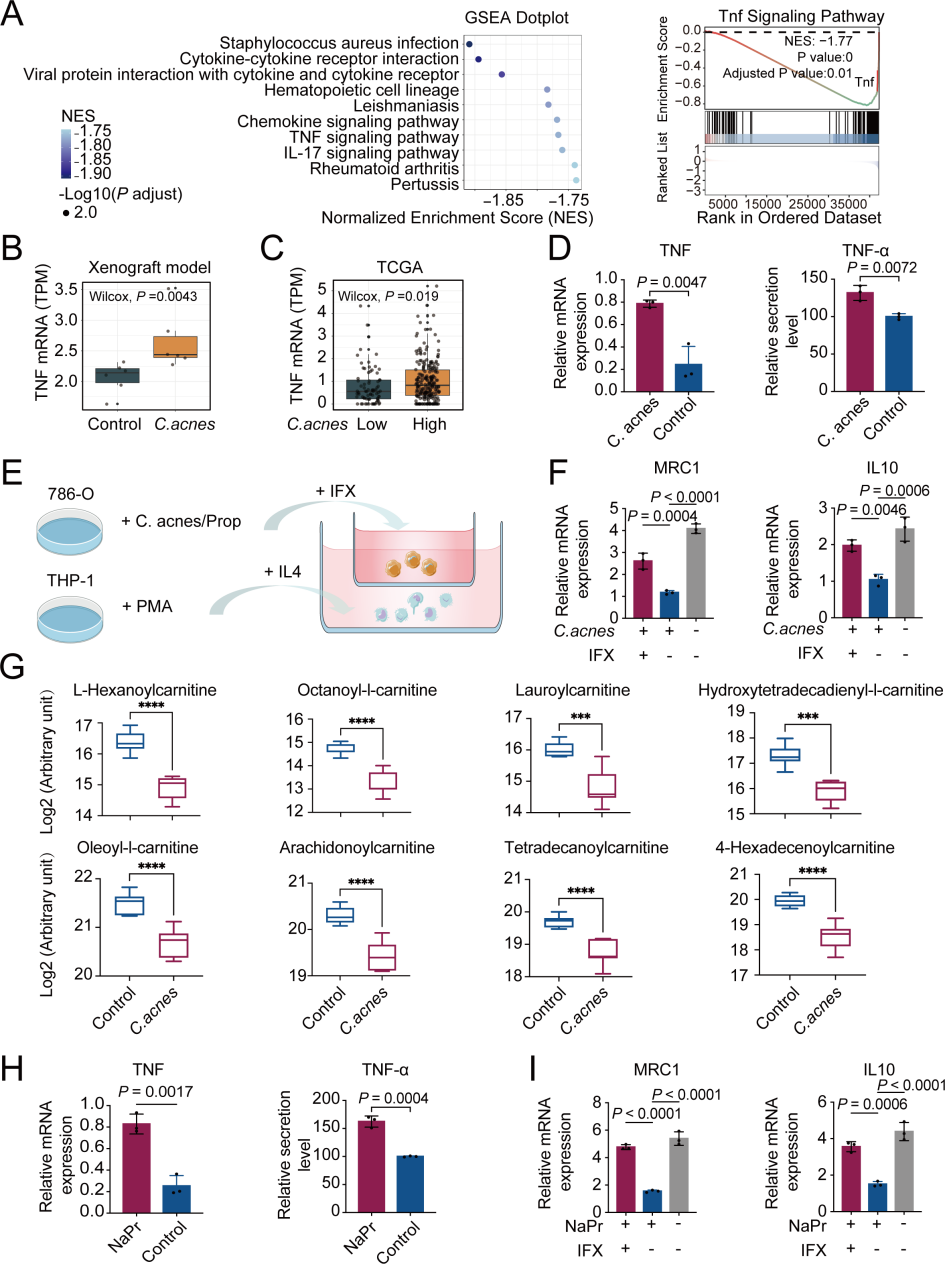
*C. acnes* suppresses macrophage M2 polarization via stimulating TNF. (**A**) Dot plot showing the top 10 enriched pathways from the gene set enrichment analysis (GSEA) of differentially expressed genes between *C. acnes*-treated tumors and the control group, alongside the GSEA plot for the TNF signaling pathway. (**B and C**) Box plots displaying the mRNA expression of TNF (in transcripts per million, TPM) in (**B**) *C. acnes*-treated tumors and (**C**) ccRCC samples with higher *C. acnes* abundance from the TCGA-KIRC data set. Wilcoxon rank-sum test provided *P*-values. (**D**) Relative TNF mRNA expression and TNF-α secretion levels in *C. acnes*-treated 786-O cells. Wilcoxon rank-sum test provided the *P*-value. (**E**) Schematic diagram of the 786-O and THP-1 co-culture system. The upper chamber contains 786-O cells treated with either *C. acnes* or propionic acid. THP-1 cells were differentiated into M0 macrophages by incubation with 320 nmol/L PMA for 18 h. Subsequently, 4 × 10⁵ THP-1 cells were seeded into the lower chamber of a 6-well transwell plate and supplemented with IL-4 (20 ng/mL) to induce M2 polarization. Finally, THP-1 cells were harvested for qPCR quantification of MRC1 and IL-10 mRNA. (**F**) Relative mRNA expression of MRC1 and IL-10 in polarized THP-1 cells co-cultured with 786-O cells treated with *C. acnes*, in the presence of IFX or a control. ANOVA test provided the *P*-value. (**G**) Murine metabolomic analysis showing changes in the levels of fatty acid derivatives that serve as substrates for propionic acid fermentation. (**H**) Relative TNF mRNA expression and TNF-α secretion levels in propionate-treated 786-O cells. Wilcoxon rank-sum test provided the *P*-value. (**I**) Relative mRNA expression of MRC1 and IL-10 in polarized THP-1 cells co-cultured with 786-O cells treated with propionic acid, in the presence of IFX or a control. ANOVA test gave the *P*-value.

To elucidate the underlying drivers behind these alterations in macrophage phenotypes caused by *C. acnes* injection, we performed functional annotation comparison between *C. acnes-*treated tumor and control groups and found a significant difference in cytokine/chemokine signali, among which TNF signaling ranked top (Fig. 5A). It has been well-established that TNF could inhibit M2 polarization of macrophage (26). Consistently, we found that TNF mRNA expression was significantly increased in *C. acnes-*treated tumors (Fig. 5B), as well as in clinical ccRCC samples with higher *C. acnes* abundance (Fig. 5C). The 786-O cell also showed higher TNF expression and TNF-α secretion when co-cultured with *C. acnes* (Fig. 5D), confirming that *C. acnes* stimulates TNF signaling. THP-1 macrophages were incubated with the supernatant from the aforementioned *C. acnes* and 786-O co-culture (Fig. 5E). Compared to controls, this treatment led to a significant decrease in the mRNA levels of MRC1 and IL-10, which are key M2 polarization biomarkers. However, this suppressive effect was reversed upon the administration of the TNF inhibitor infliximab (IFX) (Fig. 5F). This suggests that TNF stimulation is indispensable for the modulation of M2 polarization suppression induced by *C. acnes*.

We next investigated how *C. acnes* modulates host TNF expression by analyzing the metabolome of *C. acnes*-treated renal tumors. We observed a drastic reduction in fatty acid derivatives—the primary substrates for propionic acid fermentation, which is the major metabolic output of *C. acnes* (Fig. 5G). Propionic acid is known to epigenetically modulate gene expression via histone propionylation, and TNF is an upregulated target due to increased chromatin accessibility (27). As expected, propionate acid treatment significantly elevated both intracellular TNF mRNA expression within 786-O cells and the subsequent extracellular secretion of TNF-α protein into the culture media (Fig. 5H). Consistent with the effects observed with *C. acnes*, the suppression of M2 polarization markers (MRC1 and IL-10) in macrophages, which was induced by the conditioned medium from propionate-treated 786-O cells, was effectively reversed following TNF inhibitor treatment, compared to the control group (Fig. 5I). Collectively, these results demonstrate that the intratumor *C. acnes* and its metabolite, propionate, inhibit TNF-mediated macrophage M2 polarization in ccRCC.

## DISCUSSION

In this study, the presence of intratumor bacteria in ccRCC was successfully identified by multi-cohort and multi-omic data sets, along with experimental validation. Although we observed differences in specific bacterial taxa, the overall microbial diversity, composition, and putative bacterial load were comparable between cancerous and normal tissues. Leveraging the abundance of 13 genera, we constructed microbial risk scores for predicting the OS and PFS, which both demonstrated robust efficacy in prognostic stratification. Analysis of host-microbe correlation indicated that intratumor bacteria may mediate their effects on the tumor microenvironment via immune- and metabolism-related pathways. Last but not least, we isolated *C. acnes* from fresh renal tumor tissues and found that it functioned as an anti-tumor agent by suppressing macrophage M2 polarization via stimulating tumor TNF signaling. Building upon the preprint study (28), we further refined the genus-based prognostic risk scores and successfully isolated and cultured *C. acnes* directly from tumor tissues, demonstrating its tumor-suppressive effects and elucidating the underlying molecular mechanism, conducting a more accurate and comprehensive extension of the research.

Lack of difference in bacterial loads, composition, or diversity between normal and cancerous tissues is one of our major findings. Wang et al. (9) mentioned that cancerous microbiota exhibited reduced α-diversity and distinct β-diversity compared to adjacent normal tissues. However, Chen et al. (29) held the opinion that alpha-diversity was increased in ccRCC tumor tissues. Besides, Kovaleva et al. (30) proposed that the bacterial burden was higher in adjacent normal tissue than in the tumor. The results of the above contradictions indicate that more complete and reliable methods and a larger number of samples are still needed to draw more reliable conclusions. Therefore, the integration of paired samples from different data sets in our study, as well as the rigorous decontamination, makes our observations more in line with the real situation, although we cannot exclude the fact that the bacterial community drastically differs at the species or strain level upon carcinogenesis, which needs further exploration. There are also some studies showing that the composition of the bacterial community is not distinct between normal and cancerous tissues in other tumors (31, 32), supporting our conclusion. We speculate that the intratumor bacteria may originate from these normal tissues, and only certain ITB species, rather than genera, could alter in abundance in the cancer environment, supporting the “passenger” role of intratumor bacteria. When the microenvironment becomes immunosuppressed and hypoxic, microbial colonization may be easier and then contribute to the progression of tumors. In comparison to previous studies (9–11, 33), several genera appeared to be ubiquitously present at high relative abundance, such as the *Staphylococcus* and *Acinetobacter*. However, the ITB features associated with prognosis were not among the top abundant ones, further implying that ITB could be sporadic and commensal, not only in ccRCC but also in the kidney.

Our 13-genera microbial risk scores performed well in predicting the prognosis. Furthermore, the patients in the subgroups stratified by the microbial risk scores showed distinct biological characteristics, which implied that the bacterial community may influence the tumor progression via immune and metabolic pathways. In support of our conclusion, Hu et al. (34) also highlighted the correlations between prognostically relevant microorganisms and immune-related differentially expressed genes in renal cell carcinoma, and a study (30) observed a significant correlation between intratumor bacterial burden and immune cells in kidney tumors. In another study (35), researchers referred that alteration of metabolic genes may affect the intratumor microbiota in renal tumor tissues. In fact, the role of intratumor microbiota in reprogramming of cancer immune and metabolism has been increasingly recognized in lots of other tumors (23, 36–40). Notably, the genera *Rothia*, *Cutibacterium*, *Corynebacterium*, and *Actinomyces* seemed to dominate among these microbial communities across cohorts, and they were identified as diagnostic biomarkers, effective prognostic tools, and tumor progression indicators in many tumors such as hepatocellular carcinoma (41), adenoid cystic carcinomas (42), breast cancer (43), nasopharyngeal carcinoma (44), and so on. The mechanisms of how intratumor microbiota affects tumorigenesis and treatment mainly focus on the profound immunomodulatory roles and metabolic reprogramming (7, 45). It is worth mentioning that a certain microbial feature is associated with response to Nivolumab in ccRCC, in reminiscence of a recent trial modulation gut microbiome in metastatic ccRCC patients receiving Nivolumab plus ipilimumab therapy (46). The ITB community in our findings is closely related to decreased immune escape, for example, inhibition of antigen presentation and decreased M2 polarization, both showing pro-inflammatory effects. Interestingly, ITBs with different clinical associations seldom overlap, and we have not identified such an “omnipotent” ITB in ccRCC. Despite this, *Corynebacterium* is of interest as its abundance ranks top 20 in most cohorts and is associated with nivolumab response with the highest AUC. Besides, *Bifidobacterium* supplement has been shown in a trial that augments ICI response in metastatic ccRCC patients (46), and our findings that intratumor *Bifidobacterium* was protective shed light on the thus far elusive mechanism of this gut-tumor axis.

We successfully isolated and cultured *C. acnes* from renal tumor tissues, and multi-omics analyses using both *in vitro* and *in vivo* models consistently supported a protective role of *C. acnes. C. acnes* is an aerotolerant anaerobic, gram-positive bacterium predominantly residing in the sebaceous glands of human skin (47). Historically regarded as a commensal organism, it has increasingly been recognized as an opportunistic pathogen associated with inflammatory conditions such as endophthalmitis, endocarditis, osteomyelitis, sarcoidosis, keratitis, and SAPHO syndrome (48). Although *C. acnes* is often regarded as a potential contaminant, accumulating evidence has demonstrated its authentic intratumor presence across multiple malignancies, including prostate, breast, and thyroid cancers (49–52). Notably, while *C. acnes* has been implicated in tumor-promoting or immunosuppressive roles in prostate and thyroid cancers (49, 51, 52), our findings align more closely with observations in breast cancer, where *C. acnes* abundance has been associated with favorable clinical outcomes (50). These discrepancies underscore the context-dependent role of *C. acnes* in tumor biology, likely influenced by tumor type, microenvironmental features, and host immune status. Beyond renal tumors, *C. acnes* has been repeatedly detected in various solid tumors, including prostate cancer (51), nasopharyngeal cancer (53), and gastric cancer (54), where it appears to actively modulate the tumor immune microenvironment. However, *C. acnes*-induced immune responses may exert divergent effects on tumor progression. In prostate cancer (51), *C. acnes* infection has been associated with increased regulatory T cell infiltration and elevated expression of PD-L1, CCL17, and CCL18 in macrophages, potentially contributing to an immunosuppressive microenvironment. Similarly, higher intratumor abundance of *C. acnes* has been observed in radiotherapy non-responders with nasopharyngeal carcinoma, suggesting a role in immune evasion and tumor persistence (53). *In vitro* co-culture experiments further revealed that conditioned media from *C. acnes*-stimulated macrophages could enhance the migratory capacity of gastric cancer cells, a process mediated by macrophage type 2 polarization through the TLR4/PI3K/Akt signaling pathway (54). In our study, *C. acnes* has been shown to induce renal tumor cells to secrete TNF-α, thereby suppressing macrophage M2 polarization and inhibiting tumor growth, further implicating this bacterium in tissue remodeling and tumor-immune interactions. These findings collectively highlight the plasticity of *C. acnes*-driven immune modulation within different tumor contexts.

The detection and identification of intratumor microbes in ccRCC are crucial in studying the microbial composition and its role in disease progression. Compared to the previous research on intratumor bacteria (Table S11), our study encompassed thus far the largest number of ccRCC cases subjected to ITB detection, functional analysis through multi-omics integration, and experimental validation from culture to mouse model. Despite the pitfalls present in ITB studies, true bacterial signatures could still be identified by imperfect sequencing technologies and decontamination processes, which has been demonstrated through experimental validation (44, 55). When consistent findings emerge from multiple cohorts, the influence of these pitfalls can be minimized, leading to more reliable and robust conclusions. Notably, unlike Poore et al. (56), before taxonomic mapping, we not only aligned clean reads against nine mammalian genomes, including the human genome, but also incorporated indices from plasmids, vectors (UniVec), mitochondria, etc., to filter out potential contamination sources, followed by further data filtering and elimination of non-human-associated genera. Besides, the microbial abundance was assessed using either the CPM format or relative abundance to avoid the erroneous data transformation causing microbes with raw sequencing read counts of zero to generate specific, non-zero artificial values. We did not put much effort into imaging ITB. For low-biomass cancer, both LPS and FISH staining could harbor magnified signals from extra-tumor bacterial contamination (57). We consider multiple sequencing platforms together with FISH signaling adequate to prove the existence of ITB. The anatomical localization of the intratumor microbiota—whether sequestered intracellularly or within the extracellular matrix (ECM)—remains a critical point of discussion (58). Based on the technical principles of droplet-based scRNA-seq, intracellular bacteria would be co-encapsulated with their host cells (59). However, our scRNA-seq analysis yielded minimal microbial signals within host cells, initially suggesting an extracellular localization. Paradoxically, our electron microscopy (EM) analysis unequivocally identified bacterial-like structures in both intracellular and extracellular compartments. This spatial discrepancy highlights the highly heterogeneous and complex ecological niche of ITB within the tumor microenvironment, underscoring the need for further large-scale, high-resolution imaging studies to fully elucidate its precise spatial dynamics.

Our research had certain limitations. First, RNA-seq, rather than whole-genome sequencing data, was utilized to profile the intratumor microbiota; these types of data were questioned by Ge et al. (25), who considered poly(A)-based transcriptomes unable to capture microbial signals. Nevertheless, we have indeed captured effective reads, despite the very few cells compared with bulk sequencing. In fact, the “poly(A)” problem in the intratumor microbiome has also been thoroughly discussed previously (60, 61). Second, our ITB panel was only aggregated at the genus rather than the species level. We had several considerations regarding this aggregation: (i) profiling ITB reading using transcriptome and FFPE-derived data in low biomass organs was challenging, and aggregation at the species level could lead to “0” readings in too many features, and (ii) aggregation at the species level using transcriptome may cause false-positive identification of certain features. Third, the computationally derived putative microbial load should be supplemented with true bacterial load validation using qPCR. Fourth, more confounders, including the biomass of the subject (6, 62), race (63, 64), sequencing tech (65), and more rigorous decontamination protocols (66), should be considered in future studies. Furthermore, our study is limited by the absence of a standalone doxycycline-only control group in the animal models. Although the uniform administration of doxycycline across groups minimized systematic bias, the lack of a non-bacterial, antibiotic-only baseline remains a potential confounder regarding the precise extent to which the antibiotic itself modulates tumor progression and the host immune landscape. Last but not least, the causation between ITB and renal tumorigenesis remains unknown (67). Whether those microbial communities are still commensal or playing driving roles alongside tumor progression depends on human microbiota-associated murine models and microbe-phenotype triangulation (67).

In conclusion, by integrating data from multi-cohorts and multi-omic data sets, this study systematically unveiled the presence of the intratumor bacteria and its intricate associations with host immune status in ccRCC. The microbial risk scores could be used as effective prognostic tools clinically, implying the role of intratumor bacteria in tumor progression. Elucidating the mechanisms underlying how specific bacterial species and their metabolites shape the tumor immune microenvironment provides novel therapeutic targets for precision immunotherapeutic strategies.

## Data Availability

Raw 16S sequencing data have been deposited at Genome Sequence Archive under accession number CRA015452. The RNA sequencing reads are available via the Genome Sequence Archive for Human under accession number HRA006163. Metabolomic data, 16S rRNA, and whole-genome sequencing for strain identification may be provided upon reasonable request.

## SUPPLEMENTAL MATERIAL

Supplemental figures msystems.00500-26-s0001.docx Figures S1-S17.

Supplemental text msystems.00500-26-s0002.docx Supplemental methods.

Supplemental tables msystems.00500-26-s0003.xlsx Tables S1-S11.

ASM does not own the copyrights to Supplemental Material that may be linked to, or accessed through, an article. The authors have granted ASM a non-exclusive, world-wide license to publish the Supplemental Material files. Please contact the corresponding author directly for reuse.
