# Supplementary material for: A comprehensive study of intratumor bacteria in clear-cell renal cell carcinoma identifies Cutibacterium acnes as a protector via suppression of macrophage M2 polarization
Source: mSystems. 2026 Jul 15;11(8):e00500-26. doi: 10.1128/msystems.00500-26 (PMC13483239; doi:10.1128/msystems.00500-26)
Supplement: Supplementary file 1 [file msystems.00500-26-s0001.docx]

**Supplementary Figures**

[Figure S1. Study design and flowchart	2](#_Toc227750691)

[Figure S2. Bacterial genera detected in bulk RNA-seq data from six cohorts	3](#_Toc227750692)

[Figure S3. Decontamination statistics for bulk RNA-seq data from six cohorts	4](#_Toc227750693)

[Figure S4. Decontamination statistics for bulk RNA-seq data and 16S rRNA sequencing data	6](#_Toc227750694)

[Figure S5. Relative abundance of intratumor microbiota in ccRCC	8](#_Toc227750695)

[Figure S6. Single-cell RNA sequencing data analysis	10](#_Toc227750696)

[Figure S7. ITB does not differ between cancer and adjacent normal tissue in ccRCC	11](#_Toc227750697)

[Figure S8. Identifying genera differentially enriched in tumor and adjacent normal tissues	12](#_Toc227750698)

[Figure S9. Factors associated with intratumor bacteria composition in tumor tissues	14](#_Toc227750699)

[Figure S10. Association of microbiome profiles with overall survival and progression-free survival	16](#_Toc227750700)

[Figure S11. Putative ITB load is associated with progression-free survival in ccRCC	17](#_Toc227750701)

[Figure S12. Identifying prognosis-related genera	18](#_Toc227750702)

[Figure S13. The prevalence and abundance of feature genera in tumor tissues	19](#_Toc227750703)

[Figure S14. Interaction among feature genera in tumor tissues	20](#_Toc227750704)

[Figure S15. Association between feature genera and immune cell proportion and immune function score	21](#_Toc227750705)

[Figure S16. Association between feature genera and immune-related genes	22](#_Toc227750706)

[Figure S17. C. acnes does not affect the growth of ccRCC cells directly	23](#_Toc227750707)

# Figure S1. Study design and flowchart

Shown were the ccRCC cohorts included, following the decontamination for 16S rRNA and bulk RNA sequencing data and the flowchart of our study.

# Figure S2. Bacterial genera detected in bulk RNA-seq data from six cohorts

The analysis of bulk RNA-seq data from six cohorts detected a total of 545 bacterial genera. Of these, 327 were identified as non-contaminant genera, while the remaining 218 were categorized as contaminant genera.

# Figure S3. Decontamination statistics for bulk RNA-seq data from six cohorts

(A) Box plot displaying the distribution of the number of sequencing reads, bacterial reads, bacterial genera, and percentage of bacterial reads in RNA-seq data from six cohorts before decontamination. (B) The Spearman correlation between total sequencing reads and detected bacterial reads, as well as total sequencing reads and the number of detected genera. The blue line represents the fitted linear regression line, with a grey shading representing the 95% confidence interval. (C) A Venn diagram illustrating the number of detected bacterial genera before and after decontamination. (D) Pie charts showing the proportion of overall bacterial genera belonging to different potential sources before (left) and after (right) decontamination, as well as the proportion of remaining genera belonging to common contaminants (E) before (left) and after (right) decontamination. (F) Box plots showing the distribution of the total relative abundance of genera of different potential sources in six cohorts with RNA-seq data, before (left) and after decontamination (right).

# Figure S4. Decontamination statistics for bulk RNA-seq data and 16S rRNA sequencing data

(A) Bar plots showing the total number of detected genera in six RNA-seq cohorts before and after decontamination. (B) Box plots depicting the distribution of detected genera in six RNA-seq cohorts before and after decontamination. The UpSetR plots illustrating the intersection of detected genera in six RNA-seq cohorts before (C) and after (D) decontamination. (E) Box plots showing the distribution of the percentage of discarded reads after decontamination (left), as well as the number of remaining bacterial reads before and after decontamination (right) in 16S rRNA sequencing data from Huashan cohort. (F) Box plots displaying the relative abundance of genera belonging to common contaminants before (left) and after decontamination (right) in 16S rRNA sequencing data from Huashan cohort.

# Figure S5. Relative abundance of intratumor microbiota in ccRCC

Alluvial plots of the phylum (A) and genus (B) level relative abundance of bacteria communities in seven datasets. Stacked bar plot of the phylum (C) and genus (D) level relative abundance of bacteria communities, with being stratified by normal and tumor tissues. Only the top five abundant phyla/genera in each dataset are shown. Before, before decontamination; After, after decontamination.

# Figure S6. Single-cell RNA sequencing data analysis

(A) Uniform manifold approximation and projection (UMAP) plot showing cell origins by color. Stacked bar plot illustrating the bacterial reads of bacterial phyla (B) or bacterial genera (C) detected in different cell types. Cells that cannot be assigned to a specific cell type due to low quality are grouped into “Unclear”.

# Figure S7. ITB does not differ between cancer and adjacent normal tissue in ccRCC

(A) Box plot showing the putative load (bacterial counts per million reads) between paired tumor and normal samples in Cohort 3, 4, and 5. The *P* values were given by Wilcoxon rank-sum test. (B-C) Box plot showing the alpha diversity measured by Shannon, Chao 1, Simpson indexes between (B) unpaired and (C) paired tumor and normal samples in Huashan dataset. P values were given by Wilcoxon rank-sum test. (D) Principal coordinate analysis (PCoA) for paired tumor and normal samples in Cohort 3, 4, and 5 based on the Bray–Curtis dissimilarity. The *P* values were tested by Permutational multivariate analysis of variance (PERMANOVA).

# Figure S8. Identifying genera differentially enriched in tumor and adjacent normal tissues

(A) Bar plot showing the genera with significantly differential abundance between paired tumor and normal samples in Huashan, Cohort 3, 4, and 5. The log2FC indicates the log2 transform ratio of the mean abundance in tumor and normal for each genus, and the Wilcoxon rank-sum test gave the *P* value. (B) The mean abundance of the common genera among Huashan, cohort 3, 4, and 5, in paired tumor and normal samples in above cohorts. (C) Bar plot showing the importance of each genus in the Random Forest model. The horizontal axis represented the decrease in accuracy, and the genus with higher value measured more importantly in distinguishing tumor against normal. (E) Four-quadrant diagram showing the predicting ability of the Random Forest model. The yellow indicated the sensitivity while the green indicated the specificity.

# Figure S9. Factors associated with intratumor bacteria composition in tumor tissues

The bar plot indicating the explained variation of each factor in the interindividual variation of intratumor bacteria composition. PERMANOVA analysis with 999 permutations, based on Bray-Curtis dissimilarity, was performed to examine the effects of clinical factors on microbial communities. All *P* values were further adjusted for multiple comparisons with the FDR (false discovery rate) method. T_stage_1_2_3_4 represents patients being grouped into four groups (T stage1, 2, 3, 4); T_stage_123_4 represents patients being grouped into two groups (T stage1, 2, 3 vs T stage 4); T_stage_12_34 represents patients being grouped into two groups (T stage1, 2 vs T stage 3, 4).

# Figure S10. Association of microbiome profiles with overall survival and progression-free survival

(A-B) Heatmap showing the associations between the first five principal components (PCs) of intratumor bacteria profile and overall survival (A) and progression-free survival (B), which were examined by the Cox proportional hazard regression models. The values in heatmaps represent *P* values. before, before decontamination; after, after decontamination. (C-D) The bar plot showing the explained variation of the (C) overall survival and (D) progression-free survival in the interindividual variation of intratumor bacteria composition. PERMANOVA analysis with 999 permutations, based on Bray-Curtis dissimilarity, was performed to examine the effects of the prognosis on microbial communities. The overall survival was grouped by median value of observed year, 1-year, 3-year, and 5-year, while the progression-free survival was grouped by median value of observed month, 6-month, 12-month, and 18-month.

# Figure S11. Putative ITB load is associated with progression-free survival in ccRCC

Kaplan–Meier curves showing the progression-free survival probability of patients stratified by the putative intratumor bacterial load in Huashan, cohort 2, and 6. The *P* values were calculated using unadjusted Log-Rank test.

# Figure S12. Identifying prognosis-related genera

Shown were genera whose abundance was related to overall survival or progression-free survival separately selected by the univariate Cox proportional hazard regression model. The Venn plot showed the interaction of the result, and the union set including 13 genera was considered prognosis related genera for following analysis.

# Figure S13. The prevalence and abundance of feature genera in tumor tissues

Bar plot showing the prevalence (present at % samples) and mean relative abundance of 13 feature genera in tumor tissues across Huashan, cohort 1, 2, and 6.

# Figure S14. Interaction among feature genera in tumor tissues

(A) Network plot showed the co-occurrence pattern of the 13 feature genera. The nodes represented the genera, and the connection represents an acceptable (|r| >0.3) and significant (*P* < 0.05) correlation. The significance of correlation was evaluated by Spearman test. The ‘abs (Cor)’ indicated the absolute value of correlation value. (B) Four indexes including betweenness, closeness, degree, and eigen were included to quantize the centrality value of each genus.

# Figure S15. Association between feature genera and immune cell proportion and immune function score

(A) Heatmap showing the association between the distance matrixes of feature genera and of the immune cell proportion in tumor tissues, and the Mantel test gave the *P* value. The connection line coloured red implied significant link, and the width of the line implied the absolute value of the Mantel’s r. The association between immune cell proportion was calculated by Spearman test. (B) Heatmap showing the association between the feature genera abundance and immune function score in tumor tissues, and the Spearman test gave the *P* value.

# Figure S16. Association between feature genera and immune-related genes

Shown was the association between abundance of feature genera and expression of genes that are biomarkers of specific immune cells including CAFs, CD4 T cells, Macrophages, and NK cells in cohort 1, 2, and 6. The Spearman test gave the r and *P* value. The connection line coloured red suggest positive while the one coloured blue suggest negative association. CAFs, cancer-associated fibroblasts.

# Figure S17. *C. acnes* does not affect the growth of ccRCC cells directly

Shown was colony formation assay in ccRCC cell lines (786-O and A498) co-cultured with *C. acnes* or control. Student’s test gave the *P* value.
