# Supplementary material for: A comprehensive study of intratumor bacteria in clear-cell renal cell carcinoma identifies Cutibacterium acnes as a protector via suppression of macrophage M2 polarization
Source: mSystems. 2026 Jul 15;11(8):e00500-26. doi: 10.1128/msystems.00500-26 (PMC13483239; doi:10.1128/msystems.00500-26)
Supplement: Supplementary file 2 [file msystems.00500-26-s0002.docx]

**Supplementary Methods**

**16S rRNA sequencing data processing and analysis**

The raw sequencing data of 16S rRNA in FASTQ format underwent processing with QIIME 2 (1) (version 2023.2). Quality filtering, denoising, and chimera removal were performed using DADA2 (2), resulting in high-quality sequences that were assigned to amplicon sequence variants (ASVs). The feature table was constructed using these ASVs, and taxonomic information was annotated using a Naive Bayes classifier (3) trained with the SILVA 138 SSURef NR99 database. ASVs that could not be confidently assigned at the phylum level, as well as non-bacterial ASVs, were excluded from further analysis.

To minimize the potential impact of contaminants, we employed a previously established decontamination process (4). This involved three steps: Firstly, we used the *isNotContaminant* function in the “decontam” algorithm (5) to identify possible contaminants. This prediction was based on the difference in ASV prevalence between FFPE samples and tissue samples. Secondly, ASVs with a relative abundance greater than 0.5% in the FFPE samples were removed. Lastly, ASVs that appeared in less than 5% of the tissue samples were further eliminated to avoid contingency. Only ASVs that met these criteria were retained for downstream analysis.

**Bulk RNA sequencing data processing**

Raw RNA sequencing data from tissue samples obtained from six cohorts were acquired online. Sequencing reads were quality-controlled using fastp v0.21.1 (6), with parameters “-l 50 -5 3 -3 3”. Filtered reads that were shorter than 50 bp were discarded. To quantify human gene expression, the clean reads were aligned to the human reference genome, GRCh38.p13, available in the GENCODE database (7) using HISAT2 v2.2.1 (8). The gene expression values were quantified in transcripts per million (TPM) using StringTie v2.2.1 (9).

For profiling the intratumor bacteria from bulk RNA-seq data, clean reads were initially aligned against an indexed database to remove host or contaminant reads. This alignment was performed using bowtie2 v 2.4.5 with a “--very-sensitive” model (10). The indexed database included 9 mammalian genomes (hg38, felCat9, canFam6, mm39, rn7, rheMac10, susScr11, galGal6, bosTau9; University of California-Santa Cruz Genome Browser), 2145438 complete bacterial plasmids (PLSDB databse, v.2021_06_23_v2) (11),13705 mitochondrial genomes (NCBI RefSeq database, accessed on Aug 15, 2022), 9443 plastid sequences (NCBI RefSeq database, accessed on Aug 15, 2022), 6093 UniVec sequences (NCBI RefSeq database, accessed on Aug 15, 2022), which were considered potential sources of human habitat- or laboratory-associated or extrachromosomal sequence contaminants for taxonomic classification of microbial metagenomic sequences (12). Unmapped paired reads were then subjected to KrakenUniq v 1.0.4 (13) for taxonomic assignment using a pre-built database (14). This database includes complete microbial genomes from RefSeq, comprising 46711 bacterial genomes, 13011 viral genomes, and 604 archaeal genomes. Additionally, the database contains 246 eukaryotic pathogens, the UniVec set of standard laboratory vectors, and the GRCh38 human genome. The abundance of bacteria was evaluated at the genus level, which was deemed more accurate than the species level (13, 15).

To ensure the removal of potential false positive assignments, the bacterial genera underwent further filtration based on the following criteria: 1) the genus must contain more than 5 reads; 2) the number of duplicated k-mer must be larger than half of assigned read counts; 3) genome coverage must be larger than 1e-5. Additionally, efforts were made to distinguish the potential host of the identified genera in order to eliminate non-human-associated genera that are likely to be contaminants. To accomplish this, information regarding the isolation sources of bacteria deposited in NCBI (https://www.ncbi.nlm.nih.gov/genome/browse#!/prokaryotes/), IMG/M (https://img.jgi.doe.gov/cgi-bin/m/main.cgi), GOLD (https://gold.jgi.doe.gov/downloads), and BV-BRC (https://www.bv-brc.org/docs/quick_references/ftp.html) was gathered. For identified genera not deposited in these four databases, potential hosts were obtained through a literature search on Google Scholar. Based on the available host information, the identified genera were classified into three groups: non-human (genera not isolated from human), human-exclusive (genera exclusively associated with the human host), and mixed (genera derived from either human or other environments). Non-human-associated genera were subsequently excluded from further downstream analysis.

**Single-cell RNA sequencing data processing and analysis**

Bacterial reads obtained from scRNA-seq were annotated using a similar approach as the bulk RNA-seq data process. In summary, the paired-end sequencing reads were aligned to the indexed database using bowtie2 v2.4.5 (10). Any reads that did not align were then processed through KrakenUniq v1.0.4 (13) for identification and assignment of microbial reads.

The raw scRNA-seq data was initially processed using cellranger v 7.1.0 (16), identifying 7017 and 8467 cells in two tumor samples (GSM5222644 and GSM5222645). Subsequently, the downstream data processing was conducted using Seurat v4.9.9.9060 (17), following its tutorial. Low-quality cells, which included those with fewer than 200 genes, more than twice the median number of detected genes, and a mitochondrial gene percentage exceeding 15%, were discarded. This resulted in 3568 and 3573 high-quality cells for GSM5222644 and GSM5222645 samples, respectively. The gene expression matrix of the remaining high-quality cells was normalized, and highly variable genes were identified. The top 2000 genes were selected for further analysis. Significant principal components (top 20) were chosen for dimensionality reduction, and clusters were identified using the *FindClusters* function with a resolution of 0.5. The *FindAllMarkers* function was used to calculate differentially expressed genes (DEGs) for each cell type. Cell type annotation was performed based on the marker genes used in a previous study (18).

**16S rRNA staining for intratumor bacteria**

We performed 16S rRNA staining in 178 samples mounted on a tissue microarray (TMA) chip from the Huashan cohort. Briefly, thorough sterilization of hood, blades, and relevant instruments was carried out. Deparaffinized sections were dehydrated, and protease K was applied at room temperature. 100 μM of EUB338‐cy5 probes (sequence: 5′-GCTGCCTCCCGTAGGAGT-3′) diluted in 1 μM working solution were applied, and samples were finalized with DAPI (1:500) staining.

**Transmission electron microscopy (EM)**

A total of 20 ccRCC tissue blocks were subject to EM. Fresh tissues were carefully handled immediately after surgical removal. Blocks sliced 1 mm³ in size were placed in a culture dish containing an electron microscope fixation solution. Samples were rinsed in 0.1M phosphate buffer (PB, pH 7.4). Samples were then placed at room temperature for 2 hours using 1% osmium tetroxide prepared in 0.1 M phosphate buffer (PB, pH 7.4). Gradual dehydration was applied, and infiltration was conducted in a mixture of propylene oxide and Epon 812 resin (1:1) at 37 °C overnight. Samples were inserted into an embedding mold filled with pure Epon 812 resin. The embedding mold underwent polymerization in 60 °C oven for 48 hours. Ultrathin sections (70 nm) were cut from resin blocks using an ultramicrotome and placed on 200 mesh Formvar-coated copper grids. Copper grids with sections were stained in a 2% uranyl acetate-saturated alcoholic solution for 15 minutes. Following three rinses with ultrapure water, sections were stained with a lead citrate solution for 10 minutes. Copper grid sections were air-dried at room temperature overnight in a copper grid box. The grids were observed under a transmission electron microscope (HITACHI, HT7800).

**Microbiome data preparation**

Microbial abundance data was converted into different formats depending on the application scenario. One is relative abundance, which refers to the proportion of a specific taxon’s abundance across different samples, and is calculated as:

$$Relative Abundance=\frac{Microbiome counts}{Total counts per sample}$$

Another format is CPM (counts per million), which could eliminate the bias introduced by sequencing depth, and is calculated as:

$$CPM=\frac{Microbiome counts\times{10}^{6}}{Total reads per sample}$$

We also proposed the putative ITB load representing the amount or concentration of bacteria present within a tumor tissue which was calculated as:

$$Putative ITB load=\frac{Total microbiome counts per sample\times{10}^{6}}{Total reads per sample}$$

For all the microbiome data in the study, the feature table, taxonomy, and phylogenetic tree after decontamination were combined into a phyloseq (19) object for downstream processing.

**Microbial diversity measurement**

The alpha diversity was measured by three indexes including Shannon, Chao1, and Simpson. The difference in the alpha diversity was compared by mean value, and Wilcoxon test gave the *P* value. The beta diversity was examined by principal coordinate analysis (PCoA) analysis based on Bray-Curtis dissimilarity which was calculated based on the relative abundance of microbial communities. Permutational multivariate analysis of variance (PERMANOVA) based on Bray-Curtis dissimilarity with 999 permutations was conducted to compare the difference in microbial communities between groups, which was performed using adonis2 in R package “vegan” (20). These groups refer to clinical parameters such as sample type, sex, age, tumor stage, survival time and so on.

**Tumor and normal tissue pairing**

To minimize potential confounding factors and enhance the reliability of our differential abundance analysis between tumor and normal tissues, we implemented a paired-sample design strategy where comparisons were strictly performed between matched tumor-normal tissue pairs from the same individual patients. This approach yielded 24, 10, 11, and 13 matched pairs in the Huashan, GSE102101, GSE126964, and GSE151419 cohorts, respectively. By employing this paired analysis framework, we effectively controlled for inter-individual variability while simultaneously addressing potential biases arising from sample collection heterogeneity and cohort-specific sample size imbalances.

**Differential abundance analysis**

To identify the genera with different abundance between tumor and normal samples, we compared the mean abundance of each group, and defined the fold change as the ratio of mean value in tumor group against that in normal group. The fold change was then log-transformed. The log_2_FC>0 indicated higher abundance in tumor samples, while log_2_FC<0 indicated higher abundance in normal samples. The Wilcoxon test gave the *P* value.

**Random forest model**

We first selected the common genera among the four cohorts as input for constructing the diagnosis-related model. The Random Forest algorithm was used to further identify the potential features distinguishing the paired tumor and normal samples using *randomForest* function in the R package "randomForest" (21). Ten-fold cross-validation and five repetitions were adopted to help select a specific number of features. The importance of each genus in the model was measured by accuracy index. We used Huashan dataset as training data, and the other datasets as validation. The predicting ability of the random forest model was measured by sensitivity and specificity, which was visualized by a four-quadrant diagram.

**Cox proportional hazard survival regression analysis**

The Cox proportional hazard regression analyses the effect of multiple factors on survival time. Hazard ratios (HR) and corresponding 95% confidence intervals (CIs) were calculated. The HR > 1 indicated that the feature was an unfavorable factor for the prognosis, while HR < 1 indicated that the feature was a favorable factor. This analysis was conducted using the *coxph* function in the “survival” package (22). The Likelihood ratio and Wald test gave the *P* values.

To explore the relationship between the microbial community and overall survival or progression-free survival, dimensionality reduction was employed to reduce the complexity of the microbial data. Principal Component Analysis (PCA) was performed using the *PCA* function in the “FactoMineR” R package (23). The first five principal components (PCs) of the intratumor microbiome PCA were retained to represent the overall intratumor microbiome, then the Cox regression analysis was employed to examine the association between each PC and prognosis.

To identify genera whose abundance is associated with survival outcomes, we performed univariate Cox regression analysis. To ensure statistical robustness, genera detected in fewer than 30% of the samples were initially excluded. Candidate genera were defined as those demonstrating consistent hazard ratio (HR) directionality in more than half of the analyzed cohorts. Ultimately, this filtering strategy yielded 13 prognosis-related genera for further investigation.

To construct the risk model on overall survival and progression-free survival respectively, we incorporated the 13 genera above and performed the multivariate Cox proportional hazard survival regression analysis. The Huashan dataset was used as training data, while the others were used for validation. The risk score was calculated as:

$$Risk Score=\sum\left( \beta i\times Mi \right)$$

where β represents the regression coefficient and M the microbial abundance.

Combined with the clinical covariates such as sex, age, tumor stage and grade, the risk score was tested using univariate and multivariate Cox to determine whether the risk score could act as an independent prognostic factor.

**The Kaplan-Meier survival curve**

Kaplan-Meier survival curves were plotted to report the association between the survival probability and clinical factors. The strategy for grouping included dichotomization of risk score (high-risk vs low-risk) and putative ITB load (high-load vs low-load). Survival curves were generated for each stratification approach, with between-group differences in survival probability assessed using the Log-rank test (significance threshold *P* < 0.05).

**Receiver operating characteristic (ROC) curve**

To evaluate the predictive performance of models, we conducted receiver operating characteristic (ROC) curve analysis. The ROC curves were generated by plotting the true positive rate (sensitivity) against the false positive rate (1-specificity) across various threshold settings. This analysis was applied to assess: (1) the risk score’s predictive capacity for both overall survival (OS) and progression-free survival (PFS), and (2) the predictive value of genus abundance in determining clinical benefit. The discriminative ability of each model was quantified using the area under the ROC curve (AUC), with higher values indicating superior predictive performance.

**Network analysis**

To identify potential hub features among the 13 prognosis-related genera, we conducted network centrality analysis. Pairwise correlations between genera were computed using the *rcorr* function from the R package “Hmisc” (24), with significance thresholds set at *P* < 0.05 and |r| > 0.3. The resulting correlation network was visualized using a circular layout for optimal interpretability.

Network centrality metrics, including betweenness (measuring brokerage potential), closeness (reflecting information propagation efficiency), degree (indicating direct connectivity), and eigenvector centrality (assessing influence within the network), were systematically evaluated to quantify the relative importance of each genus. Genera consistently ranking in the top quartile across multiple centrality indices were considered hub features, suggesting their potentially pivotal roles in the microbial community's prognostic associations.

**Differential expression analysis**

To identify differentially expressed genes (DEGs), we performed a comparative analysis using the “limma” package (25) in R. The gene expression data (TPM format) were first normalized using the voom transformation, and the linear modelling was then applied, followed by empirical Bayes moderation to stabilize variance estimates. Genes with *P* < 0.05 were considered statistically significant DEGs.

**KEGG pathway enrichment**

For functional interpretation, we conducted KEGG pathway enrichment analysis on the DEGs using the “clusterProfiler” package (26). Significantly enriched pathways were identified based on a hypergeometric test, with an adjusted *P* value (FDR < 0.05) as the threshold. The results were categorized into five types including immune, metabolism, proliferation, infection, and others based on experience and characteristics, and were visualized using density plots.

**Immune cell infiltration and immune function score assessment**

To evaluate the tumor immune microenvironment, we employed a computational immune profiling approach combining cell-type quantification and functional assessment. For immune cell infiltration analysis, we utilized the EPIC algorithm implemented in the “IOBR” package (27) to estimate the absolute proportions of eight major immune and stromal cell populations (including B cells, CD4^+^ T cells, CD8^+^ T cells, NK cells, macrophages, cancer-associated fibroblasts, endothelial cells, and other cells) from bulk RNA-Seq data, applying the default deconvolution parameters and reference signature matrix. To assess immune functional activity, we used the *gsva* function in the R package “GSVA” (28) using referenced gene sets. The resulting immune function scores were normalized across samples for comparative analysis. Both immune cell fractions and functional scores were subsequently correlated with microbial features and clinical outcomes to investigate potential microbiome-immune interactions in the tumor microenvironment.

**Mantel test and spearman correlation test**

To assess the association between microbial community composition and infiltrated immune cells, we performed a Mantel test using the *mantel_test* function in the “linkET” package (29). This non-parametric method evaluates the correlation between two dissimilarity matrices while accounting for potential spatial or temporal autocorrelation. The microbial and immune cell dissimilarity were both calculated using Euclidean distance, as the microbiome matrix was input as CPM format. We conducted the Mantel test with default permutations to assess statistical significance, with a *P* value < 0.05 as the threshold. The correlation was examined by Spearman analysis.

**Mouse models**

Sixteen female mice (18-22 g) were housed under SPF conditions (20-26 °C, 40-70% humidity, 12/12 light cycle). Renca cells (2×10^6^/mouse) were injected into the right flank; treatment began when tumors reached 50-100 mm³. Mice were pretreated with doxycycline (1 mg/mL in water, 5 days) and randomized into two groups (N = 8 per group): 1) PBS control (50 µL, intratumor, every 3 days) or 2) *C. acnes* (2×10^8^ CFU/50 µL, intratumor, every 3 days). Doxycycline treatment was used to first eliminate intratumor microbiota for 5 days when tumors reached 5 mm in diameter (30). Tumor volume (V = 0.5×a×b^2^), weight, and survival were monitored. Endpoints included tumor size > 2000 mm³ or > 20% weight loss. TGI and T/C ratios were calculated to assess efficacy.

**Colony formation assay**

After the specified treatments, cells were fixed with 1 mL of 4% paraformaldehyde per well for 30-60 min at room temperature. Subsequently, the colonies were stained with 1,000 μL of 0.1% crystal violet (Sangon Biotech) for 10-20 min. After washing and air-drying, colonies containing more than 50 cells were manually counted or quantified using ImageJ software. The experiments were performed in triplicate.

**RNA sequencing**

Total RNA was extracted from tissues using TRIzol Reagent, followed by quality assessment via NanoDrop 2000 (OD260/280: 1.8-2.2) and Agilent 5300 Bioanalyzer (RIN ≥ 6.5). For standard libraries, poly(A)-enriched mRNA was fragmented (~300 bp), reverse-transcribed into cDNA (Illumina^®^ Stranded mRNA Prep) and sequenced on the DNBSEQ-T7 platform (PE150). For low-input samples (10 ng RNA), SMART-Seq_V4 was used for cDNA synthesis and Tn5-based fragmentation. Raw reads were processed with fastp (adapter trimming, Q20 filtering), aligned to a reference genome (HISAT2), and quantified (RSEM). Differential expression analysis (DESeq2/DEGseq; |log2FC| ≥ 1, FDR < 0.05) and functional enrichment (GO/KEGG) were performed. Alternative splicing events were identified using rMATS.

**Metabolomics**

The non-targeted metabolomics analysis was performed using LC-MS (UHPLC-Exploris240 system, Thermo Scientific). Samples (50±5 mg) were homogenized in methanol: water (4:1) containing internal standards (e.g., L-2-chlorophenylalanine) using a cryogenic grinder (-10 °C, 50 Hz), followed by ultrasonic extraction (5 °C, 40 kHz) and centrifugation (13,000 g, 4 °C). The supernatant was analyzed via UHPLC (ACQUITY UPLC HSS T3 column; 40 °C) with gradient elution (mobile phases: 0.1% formic acid in water/acetonitrile and isopropanol/acetonitrile/water). MS detection employed ESI±modes (m/z 70-1050, resolution 60,000), with QC samples interspersed to monitor stability. Data were processed using Progenesis QI v3.0 for peak alignment, metabolite identification (HMDB, Metlin), and z-score-based QC validation.

**Whole genome sequencing**

Genomic DNA was extracted and the integrity and purity were determined using 1% agarose gel. After the genome DNA test, the equencing library was prepared according to the experimental report of SQK-LSK109 sequencing kit (Oxford Nanopore Technology). Library quality was evaluated using Qubit 4.0 (Thermo Fisher Scientific, Waltham, USA), and average fragment size was evaluated using Agilent 4200 (Agilent, Santa Clara, CA). Finally, sequencing was performed using Nanopore. The raw data obtained by sequencing was subjected to data filtering, then the filtered data was used for genome assembly via Unicycler (<https://github.com/rrwick/Unicycler>). The Arrow software was utilized to polish the assembly results and correct misassembled regions.

**Immunohistochemical staining for *Cutibacterium acnes***

Totally 20 formalin-fixed paraffin-embedded (FFPE) sections were deparaffinized and subjected to heat-induced antigen retrieval. To identify intratumor bacteria, sections were incubated with a primary antibody against *Cutibacterium acnes* (MBL Beijing Biotech Co.,Ltd., Catalog #D371-3) overnight at 4 °C, followed by treatment with a biotinylated secondary antibody and DAB staining. Negative control (secondary antibody only) was used to ensure specificity. Staining was evaluated by two blinded pathologists; cases with distinct brown granular staining within tumor tissue were deemed positive, and the positive rate was calculated as (positive cases/total cases) ×100%.

**Co-culture and cell treatment**

Bacterial cultures were diluted at a final concentration of 1.0 Multiplicity of Infection (MOI) for co-culture with 786-O cells. As *C. acnes* was anaerobic, we depleted oxygen at the time of co-culture. The mixture was incubated for 60 hours, and cells were subjected to qPCR. Cell viability did not alter for 60 h after co-culture. Infliximab (10 μg/mL, IFX) is a chimeric monoclonal IgG1 antibody that specifically binds to TNF-α, and it was used to prevent the interaction of TNF-α with TNF-α receptor (TNFR1 and TNFR2) (31). The supernatant was subjected to human TNF detection using ELISA kit. Sodium propionate was used as a propionate source in cell treatment. Human monocytic cells (THP-1 cells) were used in this experiment, and they were differentiated into M0 macrophages by incubating in 320 nmol/L PMA for 18 hours, then 4×10^5^ of THP-1 cells were seeded in the lower chamber of 6-well transwell plates with final IL-4 (20 ng/mL) for generating M2-polarized macrophages. The 786-O-treated supernatant was added to the upper chamber and incubated for 48 hours, finally THP-1 cells were harvested for mRNA test for MRC1 and IL10 using qPCR. The PMA, IL-4, infliximab, and sodium propionate were obtained from Medchemexpress.

**Statistical analysis**

All data analyses were conducted via R Studio software unless otherwise specified. Statistical significance was evaluated using Spearman's correlation for continuous variables and Wilcoxon rank-sum test for group comparisons, with multiple testing correction via the Benjamini-Hochberg method (FDR < 0.05) (32). In this study, we used the following notation to indicate the significance levels of *P* values: NS (*P* > 0.05), *0.01 < *P* < 0.05, **0.001 < *P* < 0.01, and *** *P* < 0.001. Data are expressed as mean ± SEM unless otherwise stated.

**References:**

1.	Bolyen E, Rideout JR, Dillon MR, Bokulich NA, Abnet CC, Al-Ghalith GA, Alexander H, Alm EJ, Arumugam M, Asnicar F, Bai Y, Bisanz JE, Bittinger K, Brejnrod A, Brislawn CJ, Brown CT, Callahan BJ, Caraballo-Rodriguez AM, Chase J, Cope EK, Da Silva R, Diener C, Dorrestein PC, Douglas GM, Durall DM, Duvallet C, Edwardson CF, Ernst M, Estaki M, Fouquier J, Gauglitz JM, Gibbons SM, Gibson DL, Gonzalez A, Gorlick K, Guo J, Hillmann B, Holmes S, Holste H, Huttenhower C, Huttley GA, Janssen S, Jarmusch AK, Jiang L, Kaehler BD, Kang KB, Keefe CR, Keim P, Kelley ST, Knights D, Koester I, Kosciolek T, Kreps J, Langille MGI, Lee J, Ley R, Liu YX, Loftfield E, Lozupone C, Maher M, Marotz C, Martin BD, McDonald D, McIver LJ, Melnik AV, Metcalf JL, Morgan SC, Morton JT, Naimey AT, Navas-Molina JA, Nothias LF, Orchanian SB, Pearson T, Peoples SL, Petras D, Preuss ML, Pruesse E, Rasmussen LB, Rivers A, Robeson MS, Rosenthal P, Segata N, Shaffer M, Shiffer A, Sinha R, Song SJ, Spear JR, Swafford AD, Thompson LR, Torres PJ, Trinh P, Tripathi A, Turnbaugh PJ, Ul-Hasan S, van der Hooft JJJ, Vargas F, Vazquez-Baeza Y, Vogtmann E, von Hippel M, Walters W, Wan Y, Wang M, Warren J, Weber KC, Williamson CHD, Willis AD, Xu ZZ, Zaneveld JR, Zhang Y, Zhu Q, Knight R, Caporaso JG. 2019. Reproducible, interactive, scalable and extensible microbiome data science using QIIME 2. Nat Biotechnol 37:852–857.

2.	Callahan BJ, McMurdie PJ, Rosen MJ, Han AW, Johnson AJ, Holmes SP. 2016. DADA2: High-resolution sample inference from Illumina amplicon data. Nat Methods 13:581–3.

3.	Bokulich NA, Kaehler BD, Rideout JR, Dillon M, Bolyen E, Knight R, Huttley GA, Gregory Caporaso J. 2018. Optimizing taxonomic classification of marker-gene amplicon sequences with QIIME 2’s q2-feature-classifier plugin. Microbiome 6:90.

4.	Sun L, Ke X, Guan A, Jin B, Qu J, Wang Y, Xu X, Li C, Sun H, Xu H, Xu G, Sang X, Feng Y, Sun Y, Yang H, Mao Y. 2023. Intratumoural microbiome can predict the prognosis of hepatocellular carcinoma after surgery. Clinical and Translational Medicine 13:e1331.

5.	Davis NM, Proctor DM, Holmes SP, Relman DA, Callahan BJ. 2018. Simple statistical identification and removal of contaminant sequences in marker-gene and metagenomics data. Microbiome 6:226.

6.	Chen S, Zhou Y, Chen Y, Gu J. 2018. fastp: An ultra-fast all-in-one FASTQ preprocessor. Bioinformatics 34:i884–i890.

7.	Frankish A, Diekhans M, Ferreira A-M, Johnson R, Jungreis I, Loveland J, Mudge JM, Sisu C, Wright J, Armstrong J, Barnes I, Berry A, Bignell A, Carbonell Sala S, Chrast J, Cunningham F, Di Domenico T, Donaldson S, Fiddes IT, García Girón C, Gonzalez JM, Grego T, Hardy M, Hourlier T, Hunt T, Izuogu OG, Lagarde J, Martin FJ, Martínez L, Mohanan S, Muir P, Navarro FCP, Parker A, Pei B, Pozo F, Ruffier M, Schmitt BM, Stapleton E, Suner M-M, Sycheva I, Uszczynska-Ratajczak B, Xu J, Yates A, Zerbino D, Zhang Y, Aken B, Choudhary JS, Gerstein M, Guigó R, Hubbard TJP, Kellis M, Paten B, Reymond A, Tress ML, Flicek P. 2019. GENCODE reference annotation for the human and mouse genomes. Nucleic Acids Res 47:D766–D773.

8.	Kim D, Paggi JM, Park C, Bennett C, Salzberg SL. 2019. Graph-based genome alignment and genotyping with HISAT2 and HISAT-genotype. Nat Biotechnol 37:907–915.

9.	Pertea M, Pertea GM, Antonescu CM, Chang T-C, Mendell JT, Salzberg SL. 2015. StringTie enables improved reconstruction of a transcriptome from RNA-seq reads. Nat Biotechnol 33:290–295.

10.	Langmead B, Salzberg SL. 2012. Fast gapped-read alignment with bowtie 2. Nat Methods 9:357–359.

11.	Schmartz GP, Hartung A, Hirsch P, Kern F, Fehlmann T, Müller R, Keller A. 2022. PLSDB: Advancing a comprehensive database of bacterial plasmids. Nucleic Acids Res 50:D273–D278.

12.	Nakatsu G, Zhou H, Wu WKK, Wong SH, Coker OO, Dai Z, Li X, Szeto C-H, Sugimura N, Lam TY-T, Yu AC-S, Wang X, Chen Z, Wong MC-S, Ng SC, Chan MTV, Chan PKS, Chan FKL, Sung JJ-Y, Yu J. 2018. Alterations in enteric virome are associated with colorectal cancer and survival outcomes. Gastroenterology 155:529-541.e5.

13.	Breitwieser FP, Baker DN, Salzberg SL. 2018. KrakenUniq: Confident and fast metagenomics classification using unique k-mer counts. Genome Biol 19:198.

14.	Gihawi A, Ge Y, Lu J, Puiu D, Xu A, Cooper CS, Brewer DS, Pertea M, Salzberg SL. 2023. Major data analysis errors invalidate cancer microbiome findings. mBio 14:e01607-23.

15.	Wood DE, Salzberg SL. 2014. Kraken: Ultrafast metagenomic sequence classification using exact alignments. Genome Biol 15:R46.

16.	Zheng GXY, Terry JM, Belgrader P, Ryvkin P, Bent ZW, Wilson R, Ziraldo SB, Wheeler TD, McDermott GP, Zhu J, Gregory MT, Shuga J, Montesclaros L, Underwood JG, Masquelier DA, Nishimura SY, Schnall-Levin M, Wyatt PW, Hindson CM, Bharadwaj R, Wong A, Ness KD, Beppu LW, Deeg HJ, McFarland C, Loeb KR, Valente WJ, Ericson NG, Stevens EA, Radich JP, Mikkelsen TS, Hindson BJ, Bielas JH. 2017. Massively parallel digital transcriptional profiling of single cells. Nat Commun 8:14049.

17.	Butler A, Hoffman P, Smibert P, Papalexi E, Satija R. 2018. Integrating single-cell transcriptomic data across different conditions, technologies, and species. Nat Biotechnol 36:411–420.

18.	Yu Z, Lu W, Su C, Lv Y, Ye Y, Guo B, Liu D, Yan H, Mi H, Li T, Zhang Q, Cheng J, Mo Z. 2021. Single-cell RNA-seq identification of the cellular molecular characteristics of sporadic bilateral clear cell renal cell carcinoma. Front Oncol 11:659251.

19.	McMurdie PJ, Holmes S. 2013. phyloseq: An R package for reproducible interactive analysis and graphics of microbiome census data. PLoS One 8:e61217.

20.	Oksanen J, Simpson GL, Blanchet FG, Kindt R, Legendre P, Minchin PR, O’Hara RB, Solymos P, Stevens MHH, Szoecs E, Wagner H, Barbour M, Bedward M, Bolker B, Borcard D, Borman T, Carvalho G, Chirico M, De Caceres M, Durand S, Evangelista HBA, FitzJohn R, Friendly M, Furneaux B, Hannigan G, Hill MO, Lahti L, Martino C, McGlinn D, Ouellette M-H, Ribeiro Cunha E, Smith T, Stier A, Ter Braak CJF, Weedon J. 2001. vegan: Community ecology package.

21.	Breiman L. 2001. Random forests. Mach Learn 45:5–32.

22.	Therneau TM. 2001. survival: Survival analysis.

23.	Lê S, Josse J, Husson F. 2008. **FactoMineR** : An *R* package for multivariate analysis. J Stat Soft 25.

24.	Harrell Jr FE. 2003. Hmisc: Harrell miscellaneous.

25.	Ritchie ME, Phipson B, Wu D, Hu Y, Law CW, Shi W, Smyth GK. 2015. limma powers differential expression analyses for RNA-sequencing and microarray studies. Nucleic Acids Res 43:e47.

26.	Xu S, Hu E, Cai Y, Xie Z, Luo X, Zhan L, Tang W, Wang Q, Liu B, Wang R, Xie W, Wu T, Xie L, Yu G. 2024. Using clusterProfiler to characterize multiomics data. Nat Protoc 19:3292–3320.

27.	Zeng D, Fang Y, Qiu W, Luo P, Wang S, Shen R, Gu W, Huang X, Mao Q, Wang G, Lai Y, Rong G, Xu X, Shi M, Wu Z, Yu G, Liao W. 2024. Enhancing immuno-oncology investigations through multidimensional decoding of tumor microenvironment with IOBR 2.0. Cell Rep Methods 4:100910.

28.	Hänzelmann S, Castelo R, Guinney J. 2013. GSVA: Gene set variation analysis for microarray and RNA-seq data. BMC Bioinformatics 14:7.

29.	Huang H. 2021. linkET: Everything is Linkable.

30.	Fu A, Yao B, Dong T, Chen Y, Yao J, Liu Y, Li H, Bai H, Liu X, Zhang Y, Wang C, Guo Y, Li N, Cai S. 2022. Tumor-resident intracellular microbiota promotes metastatic colonization in breast cancer. Cell 185:1356-1372.e26.

31.	Méndez-García LA, Trejo-Millán F, Martínez-Reyes CP, Manjarrez-Reyna AN, Esquivel-Velázquez M, Melendez-Mier G, Islas-Andrade S, Rojas-Bernabé A, Kzhyshkowska J, Escobedo G. 2018. Infliximab ameliorates tumor necrosis factor-alpha-induced insulin resistance by attenuating PTP1B activation in 3T3L1 adipocytes in vitro. Scand J Immunol 88:e12716.

32.	Benjamini Y, Drai D, Elmer G, Kafkafi N, Golani I. 2001. Controlling the false discovery rate in behavior genetics research. Behav Brain Res 125:279–284.
